# Admission Dehydration Is Associated With Significantly Lower In-Hospital Mortality After Intracerebral Hemorrhage

**DOI:** 10.3389/fneur.2021.637001

**Published:** 2021-03-08

**Authors:** Bin Gao, Hongqiu Gu, Wengui Yu, Shimeng Liu, Qi Zhou, Kaijiang Kang, Jia Zhang, Zixiao Li, Xingquan Zhao, Yongjun Wang

**Affiliations:** ^1^Department of Neurology, Beijing Tiantan Hospital, Capital Medical University, Beijing, China; ^2^China National Clinical Research Center for Neurological Diseases, Beijing, China; ^3^Department of Neurology, University of California, Irvine, Irvine, CA, United States; ^4^Research Unit of Artificial Intelligence in Cerebrovascular Disease, Chinese Academy of Medical Sciences, Beijing, China

**Keywords:** intracranial hemorrhage, dehydration, mortality, blood urea nitrogen, creatinine

## Abstract

**Background and Purpose:** Our aim was to investigate the frequency of dehydration at admission and associations with in-hospital mortality in patients with intracerebral hemorrhage (ICH).

**Methods:** Data of consecutive patients with ICH between August 2015 and July 2019 from the China Stroke Center Alliance (CSCA) registry were analyzed. The patients were stratified based on the blood urea nitrogen (BUN) to creatinine (CR) ratio (BUN/CR) on admission into dehydrated (BUN/CR ≥ 15) or non-dehydrated (BUN/CR < 15) groups. Data were analyzed with multivariate logistic regression models to investigate admission dehydration status and the risks of death at hospital.

**Results:** A total number of 84,043 patients with ICH were included in the study. The median age of patients on admission was 63.0 years, and 37.5% of them were women. Based on the baseline BUN/CR, 59,153 (70.4%) patients were classified into dehydration group. Patients with admission dehydration (BUN/CR ≥ 15) had 13% lower risks of in-hospital mortality than those without dehydration (BUN/CR < 15, adjusted OR = 0.87, 95%CI 0.78–0.96). In patients aged <65 years, admission dehydration was associated with 19% lower risks of in-hospital mortality (adjusted OR = 0.81, 95%CI 0.70–0.94. adjusted *p* = 0.0049) than non-dehydrated patients.

**Conclusion:** Admission dehydration is associated with significantly lower in-hospital mortality after ICH, in particular, in patients <65 years old.

## Introduction

Stroke was the second leading cause of deaths and disability globally in 2017, and acute intracerebral hemorrhage (ICH) accounted for 26% of all strokes (1). Dehydration is common and associated with poor outcomes in ischemic stroke (2–4). Correlation between admission dehydration and mortality of ICH during hospitalization remains unclear. The ratio of blood urea nitrogen/creatinine (BUN/CR) ≥ 15 was considered as ideal biomarker of dehydration, especially in patients with normal kidney function (3–5). We aimed to investigate the relationship between admission dehydration and in-hospital mortality in a large ICH cohort from a multicenter prospective registry.

## Methods

### Data Availability

Data are available to researchers on request for the purpose of reproducing the results or replicating the procedure by directly contacting the corresponding authors.

### China Stroke Center Alliance Registry

This study was approved by the Human Studies Institutional Review Board of Beijing Tiantan hospital. The data of patients were prospectively collected and retrospectively analyzed from the China Stroke Center Alliance (CSCA) registry. The CSCA is a national, multicenter, hospital-based, voluntary, multifaceted intervention and continuous quality improvement (QI) initiative. This multifaceted intervention includes stroke center development, written care protocols, workshops, and a monitoring and feedback system of evidence-based performance measures. The data coordinating center of the CSCA resides at the China National Clinical Research Center for Neurological Diseases and Beijing Tiantan Hospital. The CSCA registry enrolled 1,006,798 patients diagnosed as acute ischemic stroke (AIS), transient ischemic attack (TIA), ICH, or subarachnoid hemorrhage from August 1, 2015, to July 31, 2019. Patients > 18 years of age were enrolled within 7 days of symptom onset. The CSCA registry collects data through an internet-based tool (Medicine Innovation Research Center, Beijing, China). Only in-hospital data were recorded, as follow-up data were not available. All participating hospitals in the CSCA were approved to collect data without requiring individual patient informed consent under the common rule or a waiver of authorization and exemption from their institutional review board (6). The study was performed according to the principles included in the Declaration of Helsinki.

### Patient Recruitment and Data Collection

**Figure 1:**
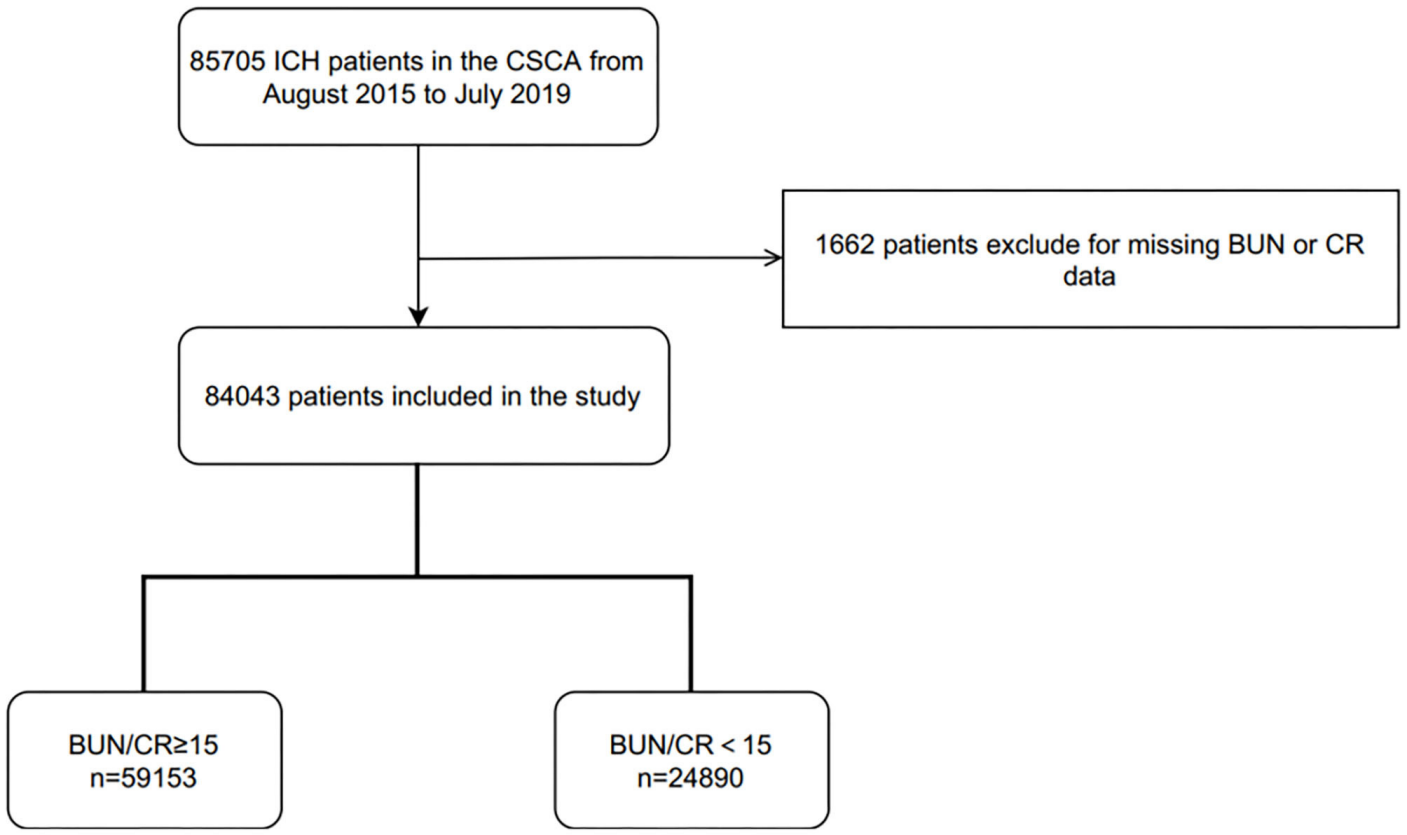
Patient flowchart. CSCA, China Stroke Center Alliance; BUN, blood urea nitrogen; CR, creatinine; BUN/CR, the ratio of blood urea nitrogen to creatinine.

Between August 2015 and July 2019, 85,705 patients with acute ICH were enrolled in the CSCA registry, and 1,662 of them were excluded for the study due to missing BUN or CR data. Therefore, 84,043 patients diagnosed with ICH were included for our study (Figure 1). ICH was diagnosed according to the World Health Organization criteria combined with imaging data by doctors in local hospital (7).

The patients were divided into dehydrated and non-dehydrated groups according to the ratio of BUN (mg/dL)/CR (mg/dl). BUN/CR ≥ 15 was defined as the dehydrated group, and BUN/CR < 15 was defined as the non-dehydrated group according to previous reports (3–5, 8).

Demographic data, stroke risk factors, and medical history, including age, sex, previous history of intracranial hemorrhage, hypertension, liver insufficiency or kidney insufficiency, current smoking and drinking, the use of antiplatelet, anticoagulation, antihypertensive, or diabetic medication, were abstracted from the registry. Laboratory test results, including fasting blood glucose, homocysteine, and admission BUN and CR levels were also extracted (Table 1).

**Table 1:** Baseline characteristics of stroke patients according to dehydration status based on the ratio of BUN/CR.

Variables^*^	**BUN/CR ≥ 15 (*n* = 59,153 [70.4%])**	**BUN/CR < 15 (*n* = 24,890 [29.6%])**	**ASD, %,^†^**
Age, years, median (IQR)	64.0 (54.0–73.0)	61.0 (51.0–70.0)	23.9
Men, *n* (%)	34,065 (57.6)	18,426 (74.0)	35.1
BMI, kg/m^2^, median (IQR)	23.5 (21.6–25.4)	23.6 (21.8–25.4)	2.1
Medical history, *n* (%)			
TIA	330 (0.6)	167 (0.7)	1.2
Cerebral infarction	7,875 (13.3)	3,080 (12.4)	2.7
SAH	339 (0.6)	117 (0.5)	1.4
ICH	10,101 (17.1)	4,575 (18.4)	3.4
Current smokers	10,638 (18.0)	5,905 (23.7)	14.1
Alcohol Consuming	13,112 (22.2)	7,446 (29.9)	17.6
Liver/renal insufficiency	478 (0.8)	768 (3.1)	16.7
Hypertension	42,081 (71.1)	17,843 (71.7)	1.3
Dyslipidemia	2,440 (4.1)	1,156 (4.6)	2.5
Atrial fibrillation	941 (1.6)	357 (1.4)	1.6
Peripheral vascular disorder	533 (0.9)	277 (1.1)	2.0
Carotid artery stenosis	213 (0.4)	93 (0.4)	0.0
Dementia	257 (0.4)	80 (0.3)	1.7
Mental disorder	251 (0.4)	70 (0.3)	1.7
Medication history, *n* (%)			
Antihypertensive	27,868 (47.1)	11,731 (47.1)	0.0
Diabetic medication	4,252 (7.2)	1,596 (6.4)	3.2
Antiplatelet	4,130 (7.0)	1,678 (6.7)	1.2
Anticoagulation	1,018 (1.7)	531 (2.1)	2.9
Cholesterol-lowering medication	3,373 (5.7)	1,517 (6.1)	1.7
Lab test results			
Homocysteine, mmol/L, median (IQR)	12.8 (9.5–17.8)	13.7 (10.0–19.4)	12.3
Fasting blood glucose, μmol/L, median (IQR)	5.9 (5.2–7.2)	5.7 (5.0–6.9)	7.0
Glycated hemoglobin, %, median (IQR)	5.6 (5.2–6.0)	5.6 (5.1–6.0)	0.0
Platelets, ^*^10^9^/L, median (IQR)	199.0 (155.0–244.0)	200.0 (156.0–245.0)	0.6

*ICH, intracranial hemorrhage; IQR, interquartile range; BMI, body mass index; TIA, transient ischemic attacks; SAH, subarachnoid hemorrhage*.

* *Continuous variables were presented as median (interquartile range), and category variables were presented as counts (percentages)*.

† *An absolute standardized difference (ASD) of >10% indicates significant differences in the variable between two groups*.

All laboratory data were the initial test results at admission. Estimated glomerular filtration rate (eGFR) were calculated by a modified four-variable Chronic Kidney Disease Epidemiology Collaboration (CKD-EPI) formula with an adjusted coefficient of 1.1 for the Chinese population to estimate eGFR (9): eGFR_CKD−EPI_ = 141 × min (CR/κ,1)^α^ × max (CR/κ,1)^−1.209^ × 0.993^Age^ × 1.018 (if female) × 1.1, where CR was creatinine, κ was 0.7 for females and 0.9 for males, α was −0.329 for females and −0.411 for males, min was the minimum of CR/κ or 1, and max indicated the maximum of CR/κ or 1. In-hospital mortality was collected as primary outcome, which was defined as all-cause death during hospitalization. In the CSCA registry, all variables and data were locally collected or adjudicated by doctors at each site.

### Statistical Analysis

Categorical variables were reported as absolute numbers with percentages, and continuous variables were reported as median along with interquartile range (IQR). We analyzed the differences in baseline characteristics between the two groups. An absolute standardized difference (ASD) of >10% indicates significant differences in the variable between two groups (10). We used ASD > 10% in univariate analysis to select out covariables that need to be adjusted for in the multivariable regression model. Covariates associated with outcomes reported in the medical literature, even if ASD < 10%, were also included in the multivariable regression model. For dichotomous outcomes, we used hierarchical binary logistic regression to determine adjusted odds ratios (aORs) and 95% confidence intervals (CIs) after median imputation of missing data (11). Missing data were minimal (≥98% complete) with the exceptions of fast blood glucose (missing in 1.2%) and homocysteine (15%). We performed group analysis for the association of BUN/CR with in-hospital mortality according to age (<65 or ≥65 years), sex, medical histories, smoking status, drinking, and renal function based on eGFR (≤60 or >60 mL/min/1.73 m^2^). All tests were two-sided, and *p*-value < 0.05 was considered statistically significant. Interaction terms were retained only when the interaction *p*-value was < 0.05. All statistical analyses were performed using SAS Version 9.4 software (SAS Institute, Cary, NC, USA).

## Results

In this large cohort study, 84,043 patients met the inclusion criteria. The median age of the patients was 63.0 years (53.0–72.0), and 37.5% of the patients were women. Among these patients, 59,153 (70.4%) were classified into the dehydration group (BUN/CR ≥ 15) and 24,890 (29.6%) into the non-dehydrated group (BUN/CR < 15) (Figure 1). Table 1 shows clinical profiles of the two groups according to hydration status at admission. Patients with dehydration at admission were older (median age, 64.0 [IQR, 54.0–73.0] vs. 61.0 [IQR, 51.0–70.0]). They were also more likely to have a history of smoking, alcohol consumption, or liver/renal insufficiency and higher homocysteine level.

The in-hospital mortality of the entire study cohort was 2.3% (1,915/84,043). It was significantly lower in the dehydration group than in the non-dehydration group (2.2% vs. 2.5%, unadjusted OR 0.86, 95%CI 0.78–0.95, *p* = 0.0029) (Table 2). Admission dehydration was associated with 13% lower in-hospital mortality after adjusting for confounders (adjusted OR = 0.87, 95%CI 0.78–0.96, *p* = 0.0050).

**Table 2:** Associations between dehydration status and in-hospital mortality and subgroup analysis.

**Group**	**No. of patients**	**No. of death/total patients (%)**		**Unadjusted**			**Adjusted** **		
		BUN/CR < 15^*^n = 24,890	**BUN/CR ≥ 15 *n* = 59,153**	**OR (95%CI)**	***p*-value**	**Interaction *p***	**OR (95%CI)**	***p*-value**	**Interaction *p***
Overall	84,043	626/24,890 (2.5)	1,289/59,153 (2.2)	0.86 (0.78–0.95)	0.0029, ^†^	/	0.87 (0.78–0.96)	0.0050, ^†^	/
**Age**									
≥65 years	38,630	296/9,922 (2.98)	800/28,708 (2.7)	0.93 0.81–1.07)	0.3094	0.011	0.97 (0.84–1.12)	0.6758	0.0211
<65 years	45,413	330/14,968 (2.20)	489/30,445 (1.61)	0.72 (0.63–0.83)	<0.0001, ^†^		0.81 (0.7–0.94)	0.0049, ^†^	
**Sex**									
Male	52,491	467/18,426 (2.53)	777/34,065 (2.28)	0.90 (0.80–1.01)	0.0685	0.448	0.89 (0.79–1.00)	0.0467, ^†^	0.8855
Female	31,552	159/6,464 (2.46)	512/25,088 (2.04)	0.83 (0.69–0.99)	0.0376, ^†^		0.83 (0.69–1.01)	0.0580	
**Hypertension**									
Yes	59,924	454/17,843 (2.54)	911/42,081 (2.16)	0.85 (0.76–0.95)	0.0044, ^†^	0.55	0.85 (0.76–0.96)	0.009, ^†^	0.4475
No	24,119	172/7,047 (2.44)	378/17,072 (2.21)	0.91 (0.75–1.09)	0.2838		0.89 (0.73–1.07)	0.2175	
**Diabetic**									
Yes	8,005	100/2,205 (4.54)	203/5,800 (3.50)	0.76 (0.60–0.97)	0.0305, ^†^	0.312	0.74 (0.57–0.95)	0.0202, ^†^	0.4352
No	76,038	526/22,685 (2.32)	1,086/53,353 (2.04)	0.88 (0.79–0.97)	0.0132, ^†^		0.90 (0.81–1.01)	0.0618	
**Current smoker**									
Yes	16,543	125/5,905 (2.12)	211/10,638 (1.98)	0.94 (0.75–1.17)	0.5601	0.385	0.91 (0.73–1.15)	0.4318	0.9158
No	67,500	501/18,985 (2.64)	1,078/48,515 (2.22)	0.84 (0.75–0.93)	0.0139, ^†^		0.86 (0.77–0.96)	0.0077, ^†^	
**Alcohol consumption**									
Yes	20,558	197/7,446 (2.65)	310/13,112 (2.36)	0.89 (0.74–1.07)	0.2113	0.759	0.90 (0.75–1.08)	0.2559	0.974
No	63,485	429/17,444 (2.46)	979/46,041 (2.13)	0.86 (0.77–0.97)	0.0111, ^†^		0.86 (0.76–0.97)	0.0136, ^†^	
**Chronic renal dysfunction**, **‡**									
Yes	8,792	355/6,001 (5.92)	171/279 (6.13)	1.04 (0.86–1.25)	0.6959	0.013	0.99 (0.81–1.21)	0.9373	0.067
No	75,251	271/18,889 (1.43)	1,118/56,362 (1.98)	1.39 (1.22–1.59)	<0.0001, ^†^		1.22 (1.06–1.40)	0.0049, ^†^	

*OR, odds ratio; BUN/CR, the ratio of blood urea nitrogen to creatinine*.

* *As reference group*.

** *Adjusted for age, male, current smokers, alcohol consuming, liver/renal insufficiency, medical history of intracranial hemorrhage (ICH), hypertension, antihypertensive, diabetic medication, antiplatelet, anticoagulation, homocysteine, and fasting blood glucose*.

† *p-value < 0.05*.

‡ *Chronic renal dysfunction defined by estimated glomerular filtration rate (eGFR) ≤ 60 mL/min/1.73 m^2^*.

There was an interaction between age and BUN/CR ratio on the in-hospital mortality (*p* = 0.0211) (Table 2). In subgroup analysis, the dehydration group was associated with significantly lower in-hospital mortality than the non-dehydration group only in patients <65 years old (1.61 vs. 2.2%, *p* < 0.0001). There was 19% lower risk of in-hospital mortality in the dehydration group (adjusted OR = 0.81, 95%CI 0.70–0.94, *p* = 0.0049). Of note, there was no difference in in-hospital mortality between the two groups in patients aged 65 or older (2.79 vs. 2.98%, *p* < 0.3094).

Further analyses of the interaction effects of sex, hypertension, diabetes, current smoking, drinking, and renal function on the association between dehydration and in-hospital mortality of patients with ICH showed that none of those interaction factors had a significant effect on the association (all of them *p* > 0.05), although the OR values for some subgroups were significant (Table 2).

## Discussion

Our study provided evidence that admission dehydration was associated with 13% lower risk of in-hospital mortality in ICH patients (adjusted OR = 0.87, 95%CI 0.78–0.96, *p* = 0.0050). Most importantly, subgroup analysis showed that admission dehydration was associated with 19% lower risk of all-cause death in hospital in patients <65 years old (adjusted OR = 0.81, 95%CI 0.70–0.94, *p* = 0.0049).

Although dehydration is a risk factor of early neurological deterioration (END) after AIS (12–14), the effect of dehydration on outcome after ICH remains unclear.

To the best of our knowledge, only one previous study focused on the relationship between admission dehydration based on the ratio of BUN/CR ≥ 15 and the prognosis of hemorrhagic stroke, and there was no difference on discharge outcomes including modified Rankin scale (mRS) and Barthel index (BI) between dehydrated (BUN/CR ≥ 15) and non-dehydrated (BUN/CR < 15) groups (3).

Different from AIS, ICH may cause immediate elevation of intracranial pressure, mass effect, and impending herniation at initial presentation. Admission dehydration might be associated with lower mass effect and herniation following ICH than the non-dehydrated cohort. We analyzed data from a large multicenter prospective registry to investigate admission dehydration and in-hospital mortality after ICH. We found that admission dehydration was associated with significantly lower in-hospital mortality, in particular, in patients <65 years old. The mechanisms of the effect are unclear. There are a few possibilities: (1) dehydration is known to increase blood viscosity and to decrease blood pressure and cerebral perfusion (4, 15), leading to reduced hematoma growth and good in-hospital outcome (16, 17). (2) Dehydration-related hypovolemia and hypernatremia may increase intravascular osmolality and reduce perihematomal edema and intracranial pressure (18–22).

This large cohort study has provided sufficient statistical power to ensure the robustness of the findings. However, our study has a few limitations. First, BUN/CR ratio is not a reliable biomarker of dehydration, in particular, in patients with congestive heart failure, gastrointestinal bleeding, or urinary tract obstruction. We were unable to exclude patients with these confounding conditions. Second, imaging data were unavailable, so it was uncertain whether the effect of BUN/CR on mortality is related to the size or location of the hematoma. Third, ICH severity on admission was not collected in our study. Last, the management of dehydration after admission was unknown. The relative benefit of admission dehydration vs. persistent dehydration remains unclear.

## Conclusions

This multicenter, large-scale prospective cohort study demonstrates the predictive value of initial hydration status on in-hospital mortality after ICH. An additional study combined with more biomarkers of dehydration, imaging data, and long-term outcomes is warranted to investigate the effect of admission dehydration on survival after ICH, in particular, in young patient population.

## Data Availability Statement

The raw data supporting the conclusions of this article will be made available by the authors, without undue reservation.

## Ethics Statement

The studies involving human participants were reviewed and approved by Human Studies Institutional Review Board of Beijing Tiantan hospital. The ethics committee waived the requirement of written informed consent for participation.

## Author Contributions

BG: designed the study and drafted the manuscript. HG, KK, JZ, QZ, and SL: major role in data acquisition and revised the manuscript for intellectual content. ZL and WY revised the manuscript for intellectual content. YW and XZ: designed and conceptualized the study, analyzed the data, and drafted and revised the manuscript. All authors contributed to the article and approved the submitted version.

## Conflict of Interest

The authors declare that the research was conducted in the absence of any commercial or financial relationships that could be construed as a potential conflict of interest.

## Abbreviations

ICH: intracerebral hemorrhage
BUN: blood urea nitrogen
CR: creatinine
BUN/CR: the ratio of blood urea nitrogen to creatinine
CSCA: China Stroke Center Alliance
eGFR: estimated glomerular filtration rate
OR: odds ratios
CI: confidence interval
IQR: interquartile range.
